# Strategies to Upgrade Animal Health Delivery in Village Poultry Systems: Perspectives of Stakeholders From Northern Ghana and Central Zones in Tanzania

**DOI:** 10.3389/fvets.2021.611357

**Published:** 2021-06-07

**Authors:** Dolapo Enahoro, Alessandra Galiè, Yakubu Abukari, Gaspar H. Chiwanga, Terra R. Kelly, Judith Kahamba, Fatihiya A. Massawe, Fausta Mapunda, Humphrey Jumba, Christoph Weber, Michel Dione, Boniface Kayang, Emily Ouma

**Affiliations:** ^1^International Livestock Research Institute, Accra, Ghana; ^2^International Livestock Research Institute, Nairobi, Kenya; ^3^Regional Department of Agriculture, Northern Regional Coordinating Council, Tamale, Ghana; ^4^Tanzania Veterinary Laboratory Agency, Mtwara, Tanzania; ^5^School of Veterinary Medicine, University of California, Davis, Davis, CA, United States; ^6^Department of Development Studies, Sokoine University of Agriculture, Morogoro, Tanzania; ^7^Department of Planning, Policy and Management, Sokoine University of Agriculture, Morogoro, Tanzania; ^8^International Livestock Research Institute, Dakar, Senegal; ^9^Department of Animal Science, University of Ghana, Legon, Ghana; ^10^International Livestock Research Institute, Kampala, Uganda

**Keywords:** poultry, value chain, newcastle disease, veterinary service, smallholder, gender, focus group discussion, qualitative analysis

## Abstract

Village chicken production holds much potential for the alleviation of malnutrition and poverty in rural communities in Africa. Owing to their subsistence nature, however, such systems are rife with infectious poultry diseases such as Newcastle disease (ND). Strategies common for the management of ND and other poultry diseases in intensive production systems, including vaccination and biosecurity measures, have seen limited success in the village production systems. New approaches are needed that can successfully deliver animal health inputs and services for the effective management of poultry health challenges in low-input systems. Our study utilized focus group discussions with men and women farmers as well as other poultry value chain actors such as input suppliers, live bird traders and processed poultry meat retailers, to investigate potential options for delivery of animal health care to village poultry systems in northern Ghana and central Tanzania. ND was commonly reported as a major disease constraint in the study sites of the two countries, with resulting fatalities particularly impactful on men and women producers and on traders. We therefore also conducted interviews that focused specifically on the gender component of village chicken production. The key health related challenges prioritized by women and men participants included limited access to, and poor quality of, vaccines and veterinary drugs, a shortage of veterinary officers, and insufficient knowledge and training of farmers on flock management practices. Women, more than men, emphasized the difficulties of accessing poultry health services. Our assessments suggest that for poultry health care delivery in the studied communities to be effective, there is need to improve the supply of good quality drugs and vaccines in rural areas, respond to the needs of both men and women, and recognize the different incentives for farmers, traders and other value chain actors. Community-based approaches and increased use of ICT technology such as mobile phones have much to offer in this regard.

## Introduction

Small-scale chicken production holds much potential for the alleviation of malnutrition and poverty in rural communities across Africa (1). However, low productivity is a major feature of village poultry production in the region, limiting capacity of smallholder poultry to deliver on its potential for addressing poverty and food security (2). Infectious poultry diseases are a key factor driving low productivity of village poultry production systems (3). In this respect, the overall inability of animal health care systems to effectively reach women farmers—observed in Tanzania and Ghana (4, 5)—makes the small-scale poultry sector, generally in the hands of women, even more vulnerable to diseases.

Although data are limited, high mortality rates in village flocks are primarily attributed to Newcastle disease (ND). ND is a highly infectious viral disease among domestic and wild birds. Virulent strains can cause up to 100% mortality among affected flocks resulting in major economic losses each year (6–8). In addition to ND, coccidiosis, fowl pox, infectious bursal disease, and less commonly avian influenza cause high morbidity and mortality in village flocks in Africa (9–11). Chickens raised in extensive production systems with minimal biosecurity measures and restricted access to veterinary inputs, including pharmaceuticals, are at increased risk of these diseases (12–15).

Disease control is difficult to carry out under free-range conditions in resource-constrained areas and is therefore limited in practice (16). Good husbandry and biosecurity practices (e.g., regular clean animal pens, quarantine new birds, isolate sick birds from the flock) provide relatively inexpensive and effective prevention measures for infectious diseases. However, most village poultry producers have not had any training on poultry keeping and there is a critical need to increase knowledge and best practices of producers. Additionally, although not always readily available to smallholder producers, vaccination offers an effective approach to specific diseases. For example, when carried out appropriately, vaccination against ND in village poultry flocks has been shown to be an effective control strategy resulting in decreased mortality and consequently increased income, utilization of poultry products, and nutrient intake among households (3, 17, 18).

In many low-input poultry systems such as the village poultry production systems, considerably more effort is needed to bridge critical gaps in policy, co-ordination, quality assurance, packaging, administration, evaluation and monitoring, training, and gender-sensitivity to facilitate successful vaccine delivery (18, 19). Such an environment could hinder investments into vaccine supply on the part of public and private agencies, as well as vaccine uptake by smallholder producers. A recent study found that while chicken-owning smallholders households place value on, and benefit from, vaccines against ND, they face substantial other barriers to vaccination (20). Studies such as (21) have highlighted the importance of market-driven approaches to addressing non-technical constraints to vaccine availability while other studies have stressed the need to understand preferences of small-scale poultry farmers and to recognize that these preferences could differ for women and men (22, 23).

Conceptually, a successful system for the delivery of animal services to village poultry value chains, at least in the context of ND management will be one that adequately addresses issues of weak effectiveness, poor availability, and inequitable access to animal health inputs (e.g., vaccines and veterinary drugs). It should also account for concerns about user perceptions and experiences of the services. The poultry value chain refers to the range of activities involved in moving product (in this case live poultry and poultry products) from the village producer to the final consumer. To be sustainable, a technically efficient animal health system serving the poultry value chain must in addition provide the right mix of incentives to relevant value-chain actors, i.e., producers, private investors and other decision-makers that critically affect its success (19). In practice, ND control programs across Asia, Africa and Latin America that have been considered technically sound and sustainable included various elements of quality control in veterinary pharmaceuticals manufacturing, field level quality assurance, the involvement of men and women farmers in program monitoring and evaluation, and active collaboration with relevant government ministries (18).

In Tanzania, constraints to development of the poultry sector are reported to include poor quality of inputs particularly veterinary drugs and vaccines, inappropriate use of veterinary drugs and vaccines and limited access of farmers to quality veterinary and extension services (24). While a locally produced vaccine (called I-2) is available that is heat stable and can withstand high temperatures (37°C), making it suitable to an environment with limited cold chain capacities; it's use is not widely established and many poultry farmers raising village breeds of chicken rely on heat-labile vaccines. Heat-labile vaccines tend to lose potency or viability if stored under unrefrigerated conditions (2–8°C) for prolonged periods (25). Electrical power shortages, non-functional and obsolete storage equipment, and inadequate temperature monitoring and control during transportation are amongst the problems constraining cold-chain vaccine delivery in rural Tanzania (26). As in many developing countries, the cold chain is usually more reliably maintained going from manufacturers to importers and distributors but becomes less so from vaccine distributors to the end-users (27).

While vaccine development and approaches to animal health care delivery in Ghana and Tanzania may over the years have better incorporated market and social considerations in their design (21, 28, 29), vaccine use is still not widely adopted in the low-resource poultry systems of both countries (30). There remains considerable ineffectiveness in the management of diseases like ND, particularly amongst producers in rural areas. To help shed light on this constraint and to assess potential solutions, our study investigated three main research questions: (1) What are the key constraints to animal health care delivery to small-scale poultry producers in northern Ghana and central Tanzania, viewed from the perspectives of the value chain actors most affected (e.g., chicken farmers and veterinary input providers); (2) How do these constraints impact on producers and others in the value chain; and (3) how does ND impact women and men farmers and other value chain actors. The responses to these questions help answer a final question: (4) What key market, institutional and other interventions could enhance the effectiveness of animal health care delivery in Ghana and Tanzania to better benefit village poultry production systems in the two countries.

The study is part of a larger study focused on development of appropriate business models to enhance the distribution of new chicken lines with improved genetics for ND resistance in village poultry production in the two countries. Breeding for resistance to viral infections is considered a viable option for addressing the ever-present threat of infectious diseases in poultry systems in Africa, given the vast genetic potential of local African chicken ecotypes (10). This approach has gathered momentum in recent years and is the focus of an ongoing research-based intervention for village poultry production in Ghana and Tanzania (30). The chicken ecotypes developed through such breeding strategies are expected to confer significantly improved but only partial resistance to ND. As such, attention still needs be paid to ways of improving animal health management and the delivery of veterinary inputs and services as part of the value chain upgrading that will be needed to support enhanced village chicken production (30, 31).

## Materials and Methods

### Value Chain Assessments

To address the research questions, we conducted assessments of the poultry value chains associated with village chicken production in selected sites in Ghana and Tanzania. Gender, participatory epidemiology, and value chain assessment frameworks were used to guide the poultry system assessments. The frameworks were operationalized through participatory appraisal methodology utilizing a value chain assessment toolkit developed under the CGIAR Research Program on Livestock (32). The toolkit provides a set of tools to analyze livestock value chains and identify, monitor, and evaluate interventions that improve value chain performance and gender inclusiveness. It has been applied widely, for instance in the Uganda pig value chains (33), in the Tanzania dairy value chain (34), and the Burundi dairy value chain (35). We applied participatory methods including pairwise ranking and value chain mapping, guided by semi-structured interview checklists adapted from the tools. Three tools from the value chain assessment toolkit were utilized.

The participatory epidemiology tool was used to identify poultry health constraints and priority diseases and their impact on poultry production systems. The value chain assessment tool was used to identify overall backyard poultry value chain constraints, map out the marketing channels for chicken, inputs and services, and document prices along the marketing channel. The gender tool was used to assess men and women's participation in the poultry value chain, identify gender-based challenges in accessing poultry inputs and markets, and identify challenges associated with poultry diseases.

### Study Area

The study was conducted in Singida and Dodoma regions in Tanzania and in Upper East region and Northern region in Ghana. The 4 regions were identified based on location of production of local chicken ecotypes, high frequency of Newcastle disease occurrence and proximity to demand areas, specifically towns such as Tamale, Bolgatanga, Dodoma and Singida. The regions are locations with growing demand for indigenous breeds of chicken. Identification of the study regions was informed by desk review and site scoping studies conducted in 2019 to identify potential target groups and poultry systems for chicken lines with enhanced ND virus resistance. The selected regions are characterized by a high population of local chicken ecotypes and households raising poultry under backyard systems.

**Figure 1:**
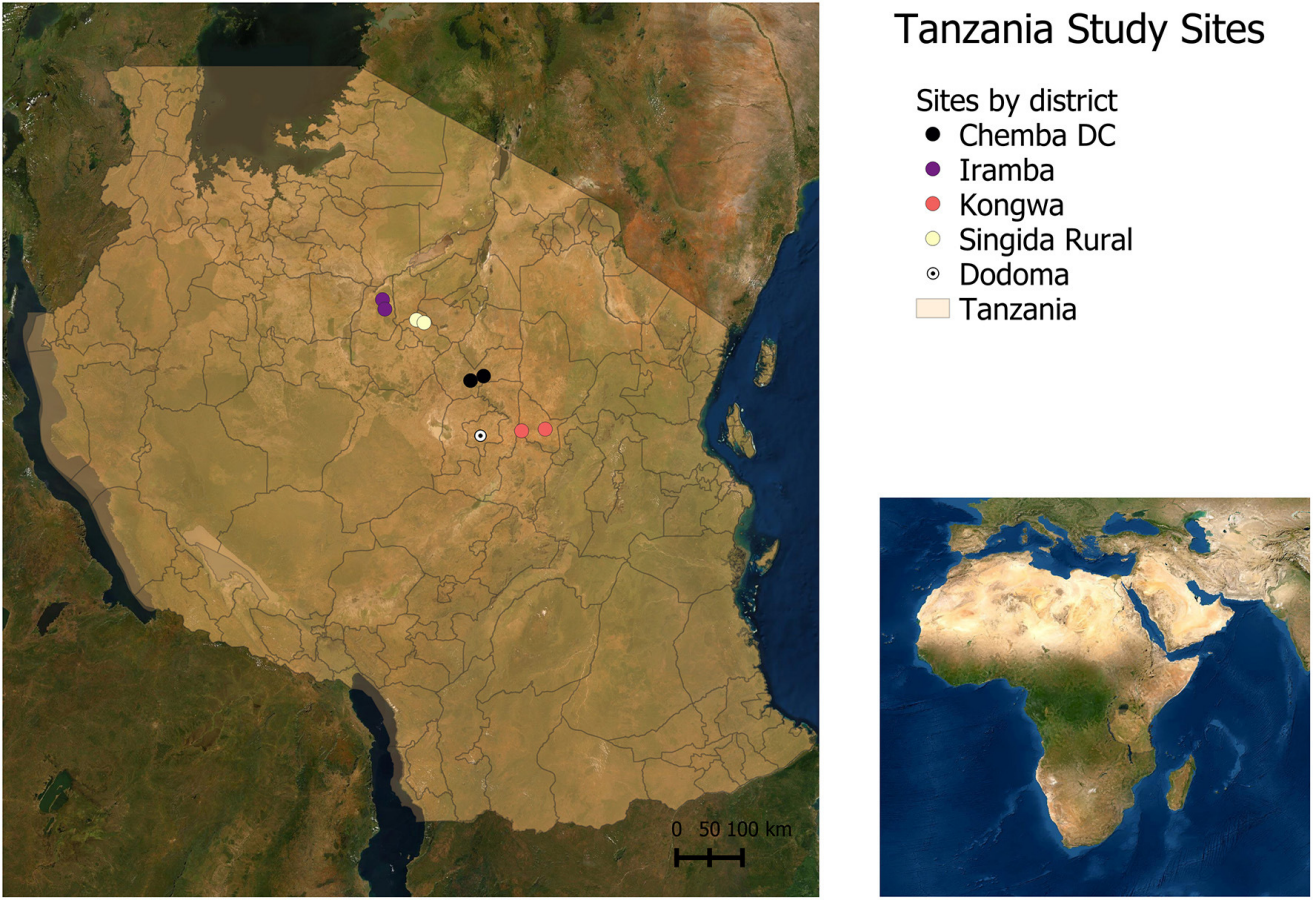
Study sites in Tanzania.

**Figure 2:**
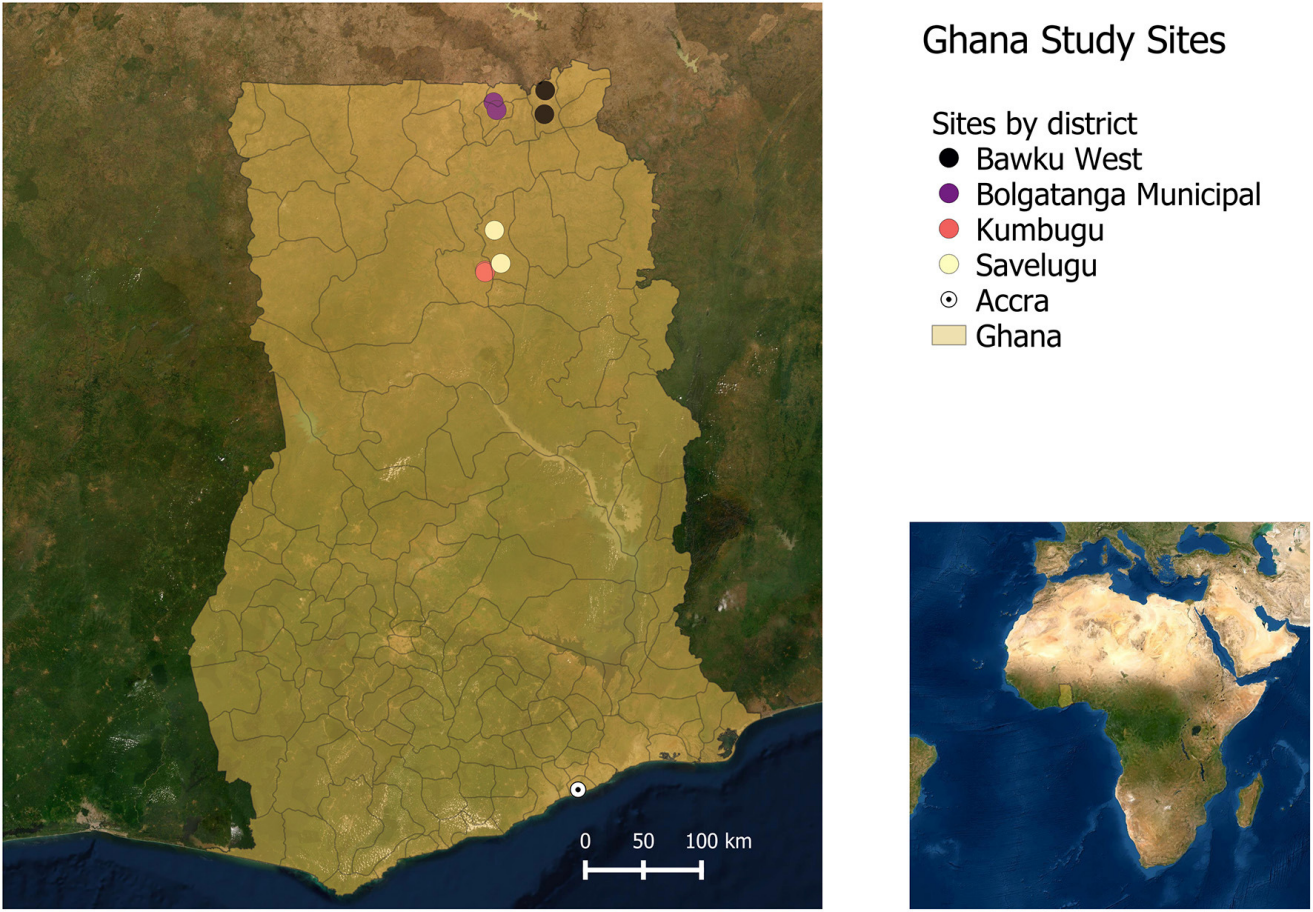
Study sites in Ghana.

In each region, two districts were selected for the value chain assessments, one representing peri urban chicken production and the other, rural chicken production that is far from urban demand centers (30). Within each district, two second level administrative divisions were selected (Metropolitan, Municipal and District Assembly, or MMDA for Ghana and ward for Tanzania), yielding a total of eight sites per country as depicted in the spatial maps in Figure 1 for Tanzania and Figure 2 for Ghana, and in Table 1. Dodoma and Singida regions are in Central Tanzania and are among the regions with high indigenous chicken population. The number of households keeping chicken in Dodoma was 139,992 in 2006, raising about 1,825,867 chickens (36). In Singida, the number of households keeping chickens was estimated at 125,895 raising 1,658,178 chickens (*ibid*.). Livestock keeping is the second major economic activity in the regions, with chicken rearing being one of the most important activities. Dodoma region has a dry savanna type of climate, which is characterized by a long dry season lasting between late April to early December and a short single wet season from January to March (37). Singida region receives rainfall from mid-November till April or early May every year (31). Temperatures in both regions range between 15^o^C and 30°C depending on season and altitude.

**Table 1:** Poultry value chain assessment sites.

**Country**	**Region**	**District**	**Site for the VCA/villages**
TANZANIA	Singida	Iramba	Old Kiomboi
			Ulemo
		Singida Rural	Mtinko
			Ikhanoda
	Dodoma	Kongwa	Sejeli/Mbande
			Kibaigwa
		Chemba DC	Gwandi
			Farkwa
GHANA	Northern	Kumbungu	Kumbungu
			Gbullung
		Savelugu	Diare
			Savelugu
	Upper East	Bolgatanga Municipal	Nyariga
			Kalbeo
		Bawku West	Zebilla
			Kukore

*Source: field work*.

Upper East Region and Northern Region throughout the study refer to administrative units in Ghana established in 1987 and bearing those names until early 2019. The Upper East and Northern regions of Ghana account for 46% of backyard chicken production, comprising indigenous chicken, guinea fowl and turkey (38). The Northern region has a chicken population of 1,744,799 and the Upper East region has a smaller population of 578,647; while the guinea fowl population is 1,414,649 in Northern region and 622,616 in Upper East region (*ibid*.). The two regions are in Northern Ghana and fall within the Northern Savanna ecological zone, with a vegetation largely comprising grasses, short woody trees, and shea and baobab trees. Daily temperatures are variable but characteristic of savanna zones, with an average daily temperature of 34°C. Some months, especially July to September are very humid. The Northern and Upper East regions are classified among the top three poorest regions in Ghana (39).

### Sample Size

In each of the four sites per region, four FGDs each comprising 12–15 participants were held with backyard chicken farmers and other poultry value chain actors. The participatory epidemiology tool was applied to one mixed-sex FGD with farmers while the value chain assessment tool was applied to one mixed sex, mixed-occupation FGD with poultry value chain actors comprising chicken traders, chicken feed traders, veterinarians, veterinary drug stockists, and chicken farmers. The gender tool was applied to two sex-disaggregated FGDs with women and men small-holder chicken farmers. The farmers and value chain actors who participated in the FGDs were randomly drawn from lists generated by the village chiefs in collaboration with agricultural extension staff in each location. For the poultry value chain actors, snowball sampling was used to identify the participants invited to the FGDs. We stopped recruiting FGD participants when we reached the principle of saturation (i.e., no new information will emerge from the discussions). A total of 64 FGDs were held, comprising 976 value chain actors (Table 2). Women made up 45 percent of all participants. The value chain assessments were held between December 2019 and May 2020.

**Table 2:** Breakdown of focus group participation by gender.

**FGD focus**	**Tanzania**	**Ghana**
**Participatory epidemiology**		
Men	71	58
Women	67	64
Gender^*^		
Men	100	115
Women	86	98
Value chain		
Men	104	92
Women	66	55
Total	494	482

* *Men and women groups were interviewed separately in the gender FGDs*.

*Source: Field work*.

### Conducting the Interviews

Each FGD was facilitated by two local enumerators who spoke the dialects of the communities and were drawn from the study regions. All enumerators were trained by experienced project scientists prior to the start of the fieldwork. The FGDs were conducted using open-ended semi-structured questions (focused on the broad topics listed previously) and in ways that allowed participants to express different opinions during the group discussions. Participants in all three FGDs were encouraged to discuss the solutions they considered relevant to addressing challenges they had identified in the smallholder poultry system. In each FGD, one enumerator facilitated the discussion and the other took written notes of the discussion. Discussions were also recorded digitally. One project scientist was present in each of the FGDs to oversee the process and ensure all emerging information was explored as appropriate. Consent was obtained by FGD participants before the start of the discussion. During the FGDs the research team provided refreshments and reimbursed participants for expenses they may have incurred to come to the meeting site.

### Data Collection

We detail here the data collection processes using the three value chain assessment tools. The results we present following are data that that the research team considered beforehand to be directly relevant to understanding the potential for improved delivery of animal health inputs and services in the village poultry systems studied. Detailed analyses of the health challenges facing the chicken production systems and of more general issues of access and delivery of health inputs and services and value chain upgrading, which also emanated from the data, are the focus of complementary results.

#### Participatory Epidemiology

The participatory epidemiology FGDs made use of proportional piling, wherein participants collectively distributed piles of (seed) counters into designated categories according to the frequencies with which they believed certain phenomena/events to occur. The method was used to elicit for communities represented in the FGDs, information on the relative importance of chicken confinement types, timings and volumes of movements in and out of local chicken flocks (e.g., through purchases and deaths) and the reasons for such events (e.g., death by disease or predation). Listing methods captured the range of husbandry practices that farmers in the area followed, while seasonality calendars were used to record participants' recall of the season occurrences of different diseases. Simple and pair-wise ranking were used to prioritize listed diseases in order of their perceived importance to chicken production in the communities. Open-ended questions were used to elicit information on the farmers' management of diseases, including vaccinations of healthy birds and disposal of bird carcasses.

#### Value Chains

The value chain FGDs included a mapping of the local chicken value chain, wherein participants collectively placed on a large blank chart the names by function, of various actors in the local chicken value chain. Markers were placed on the chart to represent the relative positioning of actors within the flow of product (chicken) through the system. Details such as geographical location and prices were then written on the chart next to the markers. For example, [live chicken, Gbullung, 20 cedis] could be written next to the marker for farmer, and [live chicken, Accra, 45 cedis] next to the marker for major live bird retailer. Participants were guided to reach consensus on where lines and arrows were to be placed that showed the relationships between actors, and direction of flow of products or services. Sub-groups of the FGD participants, by their value chain roles (e.g., traders, farmers, and veterinary input providers) identified at the start of the session, were asked function-specific questions so that, for example, farmers could respond on questions about flock mortalities while mainly traders disclosed price margins. All value chain actors were however encouraged to provide their perspectives on issues, even if these were related to nodes of the value chain different from the ones(s) they indicated that they primarily engaged in. The range of questions about the local poultry system was thus discussed among the whole group. Narratives showing both agreement and disagreement of participants within and across the sub-groups (e.g., farmers, traders, veterinary service providers) were noted.

#### Gender

Participants in the gender segregated FGDs started their sessions by collectively filling in calendars that captured the daily and seasonal time use of women or men farmers. We explored patterns of women and men participation in the poultry value chain by asking participants to detail a typical day for them, in half hour slots, and with a focus on poultry activities. We asked how this day changed over the season. We recorded main differences in a typical day among participants and explored the reasons behind these differences. The session then proceeded in a guided discussion format where the respondents discussed amongst themselves on topics related to challenges they faced in accessing inputs and markets, specific challenges brought on by poultry diseases including ND and their perceptions of the impacts of ND and other poultry diseases on different household types, and on different members within the households. To explore gender-based challenges in accessing poultry inputs and markets we asked all participants to list the main challenges women faced, describe in detail such experiences of challenges, and discuss why the challenges existed. We asked the same questions about the men. We used a similar approach when asking about challenges associated with poultry diseases.

### Data Analysis

#### Overview

Data from the focus group discussions were collected and noted in notebooks. As the data collection tools and resulting data varied somewhat in content, format, and volume (32), we analyzed outputs from the three FGD types separately. This section describes the data analysis process by focus group type. The outputs from the analyses were afterward collated and are jointly presented by theme in the results section.

#### Analyzing Data From the Participatory Epidemiology FGDs

Data in form of prioritized lists (e.g., poultry diseases) and tables (e.g., flock dynamics generated using proportional piling) were entered into excel sheets for analysis. Transcripts from the group discussions, recorded in the notes as direct responses to the survey tool's guiding questions, were similarly uploaded into excel sheets. These data were organized (e.g., using the Find, Sort, Select and File features) using Excel. The data were examined to identify emerging patterns common among women and among men, across communities and by country. Consensus was analyzed, and patterns were collated and interpreted. We indicate contradicting views that may have emerged from the discussion, when appropriate.

#### Analyzing Data From the Value Chain FGDs

Some of the data from this FGD type were captured in charts, tables and lists. These were entered into Excel sheets. The interview transcripts, which had been documented as direct responses to the survey tool's guiding questions, were also uploaded into Excel. For each question from the survey tool, individual and group responses were uploaded, and tagged such that they could be associated with specific sites, e.g., Gbullung, Ghana, and respondents, e.g., Trader T2. Responses to survey questions pre-determined to be related to disease management and the delivery of veterinary inputs were extracted and grouped by theme. These included the narratives on value chain actors' experiences and management of poultry diseases, and on access to veterinary inputs and services. The rest of the data were scanned using key word searches to identify other text relevant to the themes of interest. For example, the additional search will identify a discussion on disease impacts that may have emerged while farmers discussed their access to chicken feeds. The final collated data were then examined to identify patterns emerging from the different value chain nodes and across communities. This simple approach to collating, organizing, and analyzing the data using Excel was considered quite effective for the value chain FGDs as these had not produced voluminous amounts of qualitative data (as had the gender FGDs below, for example).

#### Analyzing Data From the Gender FGDs

The interview transcripts were uploaded into a qualitative analysis software package (NVivo Version 11). Transcripts were coded by a team of two research analysts and a gender scientist. Coding was based on a codebook developed by the team initially in a deductive manner (i.e., based on key themes from our research questions and team discussions during fieldwork). We then also conducted open coding, in which common themes that emerge from the interview notes are identified and assigned codes. Open coding allows new themes recurrently mentioned by the respondents to be captured. Discrepancies in the coding among the team members (such as length of text included under a code), were identified through NVivo, and harmonized. The coded data were examined to identify emerging patterns common among women and among men. We also checked whether other social markers, such as age, education, and marital status, could explain differences among women and among men. Consensus analysis was undertaken, and patterns were synthesized and interpreted as we present below. We indicate contradicting views that may have emerged from the discussion, when appropriate.

## Results

### Socio-Economic and Production Characteristics

The age of participants from Ghana was from 19 to 80 years, with an average of 42 years. Illiteracy level was high at the study sites in Ghana, with 60% of the participants (and 79% of women participants) having no formal education. Median age of participants in the different FGDs in Ghana was 32–52 (Table 3). The farmers mainly raised local breeds of chicken, with flock sizes ranging from 2 to 180 birds. Most of the farmers practiced free-range feeding with a few supplementing using purchased feeds. The average age of the participants from Tanzania was 42 years, ranging between 18 and 87 years old. Most of the participants (69%) had primary level education and 22% had secondary education. Median age of participants in the different FGDs in Tanzania was 28–53 (Table 4). The farmers owned local chicken breeds with flock sizes ranging between 2 and 100 birds. A few of the farmers also reared improved dual-purpose breeds of chicken on commercial basis with a flock size of more than 200 birds. Most of the farmers practiced free-range feeding with some supplementation through purchased feeds. The main constraints and impacts of the different disease management strategies are described in detail following. We report interesting within- and across-group differences in perception as they were observed.

**Table 3:** Socio-economic make up of focus group participants in Ghana^*^.

	**Kumbungu**	**Gbullung**	**Diare**	**Savelugu**	**Nyariga**	**Sherigu**	**Zebilla**	**Kukore**
**Women FGDs**								
Total #Participants	15	15	12	9	12	11	7	17
#Primary education or higher	3	3	1	1	1	5	2	4
Median age	35	42	45	52	55	34	36	40
Median flock size	16	17	23	40	13	11	25	12
**Men FGDs**								
Total #Participants	15	12	15	15	15	12	17	14
#Primary education or higher	9	5	1	6	2	2	5	5
Median age	32	38	39	40	n/a	43	46	34
Median flock size	27	68	30	40	n/a	48	27	45
**Participatory epidemiology FGDs**								
#Participants	15	15	15	16	15	15	15	17
Median age	30	30	45	35	50	40	43	42

* *Data presented are for farmer-focused groups only. Data from mixed occupation value chain FGDs are not included*.

*Data on median flock size is missing for the mixed farmer groups in Ghana*.

*Source: Field work*.

**Table 4:** Socio-economic make up of focus group participants in Tanzania^*^.

	**Old Kiomboi**	**Ulemo**	**Mtinko**	**Ikhanoda**	**Sejeli/Mbande**	**Kibaigwa**	**Gwandi**	**Farkwa**
**Women FGDs**								
Total #Participants	14	13	10	11	10	10	10	10
#Primary education or higher	14	13	10	11	10	9	10	10
Median age	42	46	42	38	40	29	44	39
Median flock size	15	10	8	10	11	11	9	8
**Men FGDs**								
Total #Participants	13	14	10	11	11	15	12	13
#Primary education or higher	12	14	10	11	10	15	12	13
Median age	53	42	28	36	35	29	46	38
Median flock size	15	17	23	10	30	11	16	10
**Participatory epidemiology FGDs**								
#Participants	16	18	14	21	15	21	18	14
Median age	48	41	32	44	40	44	42	40
Median flock size	17	14	11	22	11	15	10	11

* *Data presented are for farmer-focused groups only. Data from mixed occupation value chain FGDs are not included*.

*Source: Field work*.

### Constraints to Animal Health Care in the Village Poultry Systems

#### Presence of Poultry Diseases

In addition to ND, common poultry diseases inferred to be causing morbidity across the study sites of both countries were fowl pox, worm infestation, infectious coryza and coccidiosis. High incidences and impacts of poultry diseases were associated with limited access to veterinary service suppliers and access to veterinary products. These were due to long distances and supply shortages and led to high costs. Low quality of available products and low levels of chicken keepers' own knowledge of poultry health management were also considered major hindrances.

#### Limited Access to Veterinary Service Providers

Fifty percent of all the farmer groups in the participatory epidemiology sessions indicated shortage of veterinary service providers as a key constraint. Women also complained that their access to veterinary services had declined over time. Participants from the FGDs in Ghana were more likely than the groups in Tanzania to volunteer that they called for the services of a veterinarian to manage Newcastle or other poultry health challenges on their farms. The farmers in the Ghana FGDs also typically had larger flock sizes than those from the study sites in Tanzania (Tables 3, 4). Six (75%) of the groups in Ghana recounted calling on veterinary service providers for preventive care in form of vaccines. They reported that they called in a veterinary officer just ahead of when ND outbreaks were expected to occur. In addition, five (5) of the groups noted they called a veterinary officer to diagnose or treat their sick birds. In Tanzania, 25% reported calling or going to a veterinary officer to vaccinate birds. No group in Tanzania volunteered that they called veterinary officers to diagnose or treat sick birds.

Participants in Ghana stated there were too few officers available and responses to calls for the veterinarian were often delayed. One farmer lamented that “*we call the [veterinary] technical officers when our birds are sick. Most often, they do not respond promptly so we purchase our own medication to treat our birds*.” A veterinary officer noted that most farmers did not vaccinate their birds or did so irregularly. Late intervention could also lead to increased disease incidences and higher bird mortalities. Participants in one group in Tanzania shared their experience that majority of them vaccinated only after seeing that the birds of neighboring households were infected. According to the participants, since by this time their own birds were already likely infected, the vaccine instead accelerated chicken deaths. Participants in Ghana recounted times that veterinary officers responded to calls but declined to vaccinate as they suspected a Newcastle disease outbreak had already started. In these instances, bird owners were advised to purchase medications for treatment.

#### Low Availability of Veterinary Medicines and Other Products

In both countries, limited local availability of poultry vaccines and veterinary drugs was reported by 43% of the farmer groups involved in participatory epidemiology group sessions. In most cases the veterinary shops were in towns far from the poultry farmers. In Northern Region of Ghana, poultry farmers must travel to Tamale town to be able to purchase veterinary drugs. Women in both countries emphasized their limited access to veterinary drugs and vaccines (and other inputs) because of their limited mobility. The constraints made them dependent on their husbands or other male relatives to access inputs and veterinary services. Women in Tanzania listed agro-veterinary shops being far from their villages and therefore not accessible. Many women in Ghana stated they were not aware of shops where they could purchase veterinary inputs. In some cases, vaccines were not available even in the veterinary drug shops at the far locations. This was reported by a group from Old Kiomboi in Singida region in Tanzania. Participants in Ghana noted that the technical officers, who are government agents, did not receive government-issued supplies and privately procured vaccines to render veterinary services in rural areas they served. Some of the basic requirements for poultry disease prevention and control, such as disinfectants for use in the chicken coops, were also not available to the small-scale chicken farmers. This was reported by two groups in Upper East Region in Ghana – Nyariga and Zebilla.

#### High Costs of Veterinary Products

Women in both countries indicated the lack of cash to purchase veterinary inputs or services even when these were available locally. Issues were raised by men and women farmers regarding the cost of vaccine administration, particularly for small flock sizes. A farmer indicated that “*the vaccines are usually for a [large] number of birds and if your birds are fewer and you call the veterinary officer, he is not always willing to come*.” It was noted that the vaccines came in large packaging containing several doses and there had to be many birds available before vaccination could be done or unused vaccines could go to waste. According to the participants, large dosage packaging is uneconomical to farmers with fewer birds as they are charged more per bird for vaccines or treatment. One farmer noted paying ten times (Ghc 5.00) the usual amount (Ghc 0.50) for treating a single fowl (Ghc = Ghana Cedis; 1 US$ = 5.35 at time of study). Across the different sites in both countries, women typically owned smaller flocks than the men (Tables 3, 4), potentially making them more exposed to the higher costs imposed on smaller flock sizes.

#### Low Quality of Veterinary Products

The prevalence of poor-quality veterinary drugs and vaccines was reported in two groups in Tanzania—Ulemo and Kibaigwa—and by both women and men. The drugs and vaccines were reported as not effective as treatment measures or for enhancing immunity against diseases. Men and women participants in Tanzania reported that they sometimes lost their birds to ND even after vaccinating for the disease. The participants noted that quality assurance systems are weak, with minimal or no regulatory inspection and testing of veterinary drugs and vaccines for quality. The focus group participants also identified challenges with vaccine storage and with the vaccination process. In the case of ND, a trader in Tanzania suggested the challenge could be with how vaccines were stored by the distributors, to which an input supplier angrily responded “*I am not the one ensuring (poor) quality of vaccines but producers themselves do not store the vaccines properly*”.

#### Inadequate Knowledge of Good Husbandry Practices

In both countries, lack of farmer education on appropriate husbandry practices was reported by 71% of the groups, cutting across both countries. This was largely attributed to poor access to veterinary extension. Coupled with poor access to quality veterinary drugs, participants indicated that it resulted in indiscriminate use of drugs in the backyard poultry systems. Men and women farmers in both countries opined that they lacked knowledge on the correct veterinary drugs to use, where to get them, and how to administer them. They indicated that they also lacked knowledge on disease management and poultry management in general. Men in the gendered farmer groups in Ghana opined that women farmers had limited formal education and so suffered these constraints even more. Producers in a value chain FGD group in Tanzania conceded that they sometimes diagnosed poultry diseases by themselves and confused ND symptoms with those of typhoid. A trader lamented their exclusion from trainings that farmers obtained on poultry or poultry disease management. Participants in both countries said government livestock ministry officers or representatives from non-governmental or religious organizations had visited their villages/wards to train them on more general poultry management but these were not regular or adequate.

### Impacts of Poultry Diseases on Backyard Poultry Systems

#### Chicken Mortalities

Bird deaths were suggested as the major impact of poultry diseases in the communities represented. Both men and women farmers indicated that they could suffer total loss of their flocks. One farmer from Diare and three from Savelugu recounted experiences of losing their entire flock. However, all (8) farmers in the mixed value chain actor group at Kumbungu declared they had never suffered total bird losses since they vaccinated appropriately, following government department schedules. Bird losses were experienced by both farmers and traders. Farmers were considered most affected in the Zebilla and Kukore groups, while traders were seen to be most affected in Diare. In three of the mixed value chain actor groups, i.e., at Kumbungu, Gbullung and Savelugu, farmers and traders disagreed about which value chain actors were most impacted by disease-related mortalities, each actor group claiming they suffered the most. There seemed to be agreement in the Nyariga group that farmers and traders suffered equally from the losses. The focus groups in Tanzania also identified both producers and traders as being severely impacted by the effects of poultry diseases. Participants in four groups, i.e., Old Kiomboi, Mtinko, Ikhanoda, and Sejeli / Mbande agreed that farmers were most affected, while those in Ulemo, Kibaigwa, Gwandi and Farkwa thought traders were as affected as farmers.

#### Impacts on Farmers

Farmer losses were linked to reduced stocks and the loss of expenditures made on feeds. In addition, producers received lower prices for birds they sold when there was a disease threat (e.g., in the dry season) or known outbreak. The indirect effects were felt throughout the household since cash or in-kind receipts from bird sales are typically used to meet food expenses, the payment of children's school fees and household bills, and agricultural activities such as hiring tractors. Men participants also indicated that they used their poultry animals to acquire other animals, particularly goats through barter trade, and as dowry and gifts. Frequent bird deaths from disease, they said, made the business of raising birds unsustainable. Farmers got discouraged and did not want to continue raising birds. They were also hindered from expanding their operations. A producer in Farkwa in Tanzania noted, “*without vaccines, good feeds and medicines we cannot raise our birds commercially*.” Men farmers in Tanzania said they bore the added responsibility within the family to purchase the drugs to treat sick chickens, and when the chickens died, they still needed to provide for the family. A participant said “… *a man (breadwinner) is affected most because he is responsible for buying meat if the chickens are infected or die*.”

#### Impacts on Women Farmers

Many participants opined that women farmers were more severely impacted by the poultry disease outbreaks and threats. This was the consensus position of four of the groups of mixed value chain actors in Tanzania, i.e., Old Kiomboi, Ulemo, Sejeli/Mbande and Farkwa. The groups suggested that the women typically lacked the funds or collateral to access loans and were unable to revive their businesses after disease-related losses (Old Kiomboi). Women also bore major responsibilities for the family's expenditures on food consumption and other needs such as electricity and relied on their earnings from poultry to meet these needs. A woman farmer in Gwandi highlighted how important the incomes from poultry were to the women, stating “*over-dependence on the men for financial support might lead to conflicts ‘magomvi’.”* Women were also more directly impacted by the loss that bird deaths represented to farm inputs (chicken manure) and as a household food (protein) source. Women were said to slaughter some of the chicken to support household nutrition, particularly when funds were scarce to buy red meat. The women farmers thought their income losses in relation to poultry disease outbreaks were further compounded by the inability to negotiate live bird prices as well as the men.

#### Traders and Other Actors

Traders listed the main impacts they suffered from bird deaths as reduced earnings and disrupted businesses. Like farmers, traders could experience bird losses of 60–100% during a disease outbreak. Traders said that they lost unearned income in addition to their investments. A trader in Gbullung in Ghana lamented “*when we buy the birds and they die before we sell them, we lose both profit and capital*” It was not unusual with traders, they said, for many birds to die even before they reached the market. The high disease incidences (and impacts) were perceived to be associated with the common practice by traders in the communities of mixing birds from different sources as they were aggregated for/transported to market. The perception of one participant in Ikhanoda was that traders were not very capable of detecting the (Newcastle) disease and suffered greater losses as a result. Producers could sell off unhealthy birds to unsuspecting traders without detection. Other actors along the value chain indicated they were also affected by there being fewer and smaller sized birds available to purchase. The demand for poultry feed declined during an outbreak (affecting poultry input sellers) while prices of poultry products increased (affecting processed food retailers and consumers).

### Current Strategies to Manage Poultry Diseases or Mitigate Their Impacts

#### Filling Gaps in Veterinary Inputs and Services

Only four of eight mixed-gender participant groups in both countries, consisting of both men and women, reported that they purchased medicines or vaccines from veterinary stores. In the absence of proper veterinary drugs, both men and women producers and traders reported resort to the use of local formulations of traditional herbs such as moringa, aloe vera and pepper, as well as human drugs and food products to “treat” sick birds. Women farmers in Ghana said they used human antibiotics in lieu of vaccines for their birds while a farmer in Gwandi, Tanzania said he fed fresh milk in small quantities to chicken as medicine. Participants noted that most of the affected birds treated for suspected ND using home-based therapies still died. Men and women farmers also practiced local adaptations of biosecurity measures, including spreading ash in chicken coops to avoid the spread of disease after there had been bird deaths. A group in Tanzania made up of men farmers indicated that while the women farmers relied on traditional herbs or human drugs, they (men farmers) used veterinary drugs purchased from the shops. Although some farmers got their birds vaccinated for ND, this, by their own accounts, often did not adhere to the (regulatory government department's) guidelines.

Women from Tanzania mentioned organizing vaccination days when they would gather their small flocks and collectively use up large packages of vaccines, making them more affordable per dose/person. A men's group also in Tanzania offered that they had used social media platforms to coordinate group vaccinations for their birds. In Sherigu, Ghana, a study participant noted their use of community-based animal health personnel. Locals within the community were able to acquire some know-how and provided support to services of government veterinary and livestock officers. The producers considered this development a successful/desirable local intervention.

#### Bird Sales or Slaughter

A common practice among men and women farmers in the study communities was to sell off healthy birds ahead of the dry season or (ND) outbreak season. A male farmer in Diare opined that, given the high veterinary costs to those with fewer birds, “*the best solution sometimes is to slaughter that single fowl for household consumption*.” In one group in Ghana, all the participants indicated that they sold off most of their fowls before the onset of the season, to prevent complete losses. Some women however complained that gender norms discouraged women from fully exploiting this option. A woman from Kukore explained: “*Women in this community cannot carry their own chicken to the market to sell because it is culturally prohibited to do so. When a woman carries a chicken to the market, it can result in divorce*.” According to the women, the man could decide when to sell their wives' chickens, and controlled bird sales and proceeds from sales. The men agreed that a man could sell off his wife's chicken even if she disagreed with the sale.

As we have indicated in the discussion on impacts above, participants raised the issue of some farmers not being transparent about the health status of their flock, effectively shifting some of the potential bird losses through trade. A study participant in Farkwa however pointed out that once traders were aware of a Newcastle disease outbreak, they stopped purchasing birds in the general area and producers were left to absorb eventual losses. One group in Ghana noted that the consensus in their community was that sick birds could be slaughtered and consumed within the household, but not sold to traders or food vendors. There were no reports at any of the chicken farming communities of households slaughtering healthy birds for consumption, in anticipation of disease outbreaks.

### Interventions Suggested by the FGD Participants

#### Increase Supplies of Vaccines and Veterinary Drugs

Participants in Tanzania noted that vaccines need to be made promptly available and that this was the responsibility of the government, working through extension officers and farmer co-operatives. There was a call for vaccine manufacturers to better adhere to quality standards and on government to perform improved quality assurance. Veterinary vaccines and drugs should undergo regular inspection and testing for quality. Women farmers asked that veterinary shops be brought to the villages. According to participants, the government needed to coordinate a vaccination program to be implemented by extension officers who will provide regular farmer trainings on vaccination and more general poultry health management. Producer cooperative groups and village community meetings were some mechanisms that village chicken producers thought could be utilized to facilitate these. Some participants in both countries thought that governments or other entities should facilitate regular access, while the costs of vaccines, drugs and farmer education could be borne by them, the end users. Others suggested that veterinary drugs, vaccines, and other inputs such as strong disinfectants needed to come at subsidized prices to village poultry producers.

#### Strengthen Livestock and Veterinary Extension

In both countries, participants offered that the extension services to poultry producers needed strengthening. Men and women farmers highlighted the importance of education and training regarding vaccination and that they needed to adopt modern methods of poultry management. The establishment of demonstration farms and increased connections to farmer co-operative groups were identified as important. Respondents thought the government livestock departments needed to employ more extension and veterinary personnel, including at the ward/village level. Women farmers in Tanzania asked that extension officers be allocated permanently in each village to support farmers. This service, they opined, should extend to odd hours as they sometimes faced challenges with poultry diseases late in the night. Women farmers in Ghana also asked that community volunteers be trained to assist farmers with urgent treatments for their chickens, and that more veterinary officers be recruited to expand the coverage of farming families. Men and women respondents in Ghana sought more oversight of field officers deployed from the Ministry and asked that there be increased consultations with the communities regarding the recruitment and deployment of officers.

#### Improve Farm Management

Farmers in both countries noted that they needed training on poultry and disease management and needed to adopt improved poultry technologies. Women farmers in Tanzania and Ghana said they needed chicken housing that could better protect their birds and were easier to clean. The farmers also highlighted the needs for improved access to good chicken breeds that were resistant to disease, and to credit to purchase poultry production inputs. All farmer groups agreed they will be willing to raise birds with increased natural resistance to ND even if it did not confer total immunity. They indicated they would pay (varying amounts) more than current bird prices to access such breeds. Men farmers in Tanzania indicated their preferences for contract farming, and for better access to inputs, and to markets. Participants in Tanzania noted that government, private sector and NGOs could engage more to provide information and training to end what they identified as a patriarchal system within their society, that was present in the village poultry value chain and constrained poultry development.

## Discussion

The poultry health delivery systems we investigated in Ghana and Tanzania are known to face several constraints that limit their effectiveness. Disease-related constraints prioritized by the nearly 1,000 respondents in our study highlighted the limitations in management of infectious chicken diseases as being largely in the areas of availability, access to, and quality of vaccines and other veterinary inputs and services. These findings echo previous results for village poultry systems in Ghana (40) and Tanzania (41). Our study however provides additional context into how these constraints are experienced, differently, by diverse actors in the poultry systems and countries of the study.

### There Is Interest Among Farmers to Adopt Appropriate Technologies

While men and women farmers reported that they sometimes lost birds despite vaccinating, the overall narrative was that they got good results with proper vaccination, and expected that regular use of good quality vaccines could protect their flocks from disease (particularly ND)-related losses in the future. This result suggests that farmers perceive good vaccines to be beneficial for poultry production in their communities. It is an indication of possible interest amongst farmers to adopt vaccine technologies if they consider them of good quality. Vaccine delivery systems that will be compatible with the communities studied will however need to not only circumvent the systemic issues constraining access and quality of vaccine, but do so in manners that recognize the peculiar needs of key constituents such as poor farmers and women farmers.

Examples from East Africa, of dairy hub innovations that facilitate access of smallholder farmers to both inputs and markets, including through providing access to low-cost (appropriate) chilling technologies (42), may have lessons for improving the delivery of animal health inputs and services to the farming communities in our study.

### Increased Focus to Be Paid to Poorer Farmers and Women Farmers

Long distances and high fixed costs, arising from high transportation costs and in the case of vaccines, products that come packaged in large dose batches, mean that current limitations in access are felt much more acutely by those with fewer resources. In Tanzania, particularly, chicken producers reported needing to patronize input suppliers at far off locations to obtain vaccines and veterinary drugs. This option was available to the better resourced of the chicken farmers who could make the journey, and in the case of heat-labile products, those who could maintain the vaccine's cold chain. For most other producers, the needed inputs remained largely unavailable. Women tend to fall more into this latter category. In the communities studied, women were less likely to have the needed infrastructure (e.g., transportation) or the cash to pay for veterinary inputs and services. They were further hindered (e.g., in parts of Ghana) by cultural norms that prevent them from managing their limited resources using mechanisms (e.g., market sales) that are readily available to others. Women also seemed more adversely impacted by the system failures, as they relied more on the proceeds from poultry keeping compared to men.

### Strengthen Farmer Capacities to Influence Outcomes

Focus group participants rightly prioritized the lack of relevant education on infectious chicken diseases and poultry management more generally, as major hindrances to their poultry production. Poor training of farmers (in poultry/disease management) limits the effective use of whatever inputs and services are available or accessible. This is being addressed in some communities through farmer education programs by NGOs and others, but it seems not in a coordinated or far-reaching manner. At the Ghana sites, the tendency was for producers to seek out the direct services of (typically government-appointed) veterinary officers who were largely unavailable and had limited coverage of the rural areas. Low levels of formal education and limited access to technology (e.g., owning a mobile phone) were heightened for women at these sites, and could explain, at least partly, the high dependence of such communities on direct assistance from veterinary departments. Chicken producers with very low levels of education and/or access to technology face particularly steep challenges, as they do not have the advantage of being able, for example, to read medicine labels, or readily access poultry farm and disease management information online by themselves.

### Low Literacy Areas to Benefit From Changing Dynamics in Education

Some participants in Ghana suggested that human resources from within the rural communities (e.g., youth who typically have more formal education than their parents) could be trained to provide back-up to the services provided by veterinary officers. Although earlier work in northern Ghana had shown clear preferences of local chicken producers for government para-vets, due mainly to higher transaction costs and poorer performance of community animal health workers (40), our research will suggest an important nuance. In areas with extremely low levels of education, local personnel with formal education, if adequately trained, could immediately fill yawning gaps in the provision of animal health services that need not so much technical expertise, but the capacity on the part of the farmers to engage meaningfully with that expertise. The engagement of skilled local persons to support in community farm management and animal health delivery potentially reduces pressures on senior expertise. It could also double as opportunities for skills development and at least part-time employment for rural youth. Such local involvement will however need be conducted under close supervision of the veterinary services departments to ensure quality of services and maintain viability of the model.

### Build on Early Advances in Collective Action

Communities being able to self-coordinate to execute bulk vaccine purchases and vaccinations is already occurring and seems to hold additional promise for the future. Well-coordinated farmer cooperatives may be better suited to facilitate farmers being able to access reliable supplies of inputs and lower their current high costs. Bulk demand of vaccines, for example, could attract private sector involvement in ways that the current dispersed and uncoordinated vaccine demand from rural areas has been unable to. The high rates of attainment of basic education and widespread access to mobile telephone technologies at some of the studied communities (in Tanzania, particularly) makes for good candidates for scaling such intervention. Existing platforms of mobile technology and smart phones could be used to more readily signal, mobilize, or synchronize demand for veterinary services in village chicken production in rural areas. In the Ghana sites where rates of formal education and access to mobile phone technology were found to be extremely low, the focus of intervention could be on the empowering of target individuals and small groups within the communities, and support for the building up of key informal networks and dissemination cells around these individuals/groups. The study by (43) will suggest that the flow of professional-level knowledge by early vaccine adopters within the community has strong influences on increasing overall adoption amongst potential users. There may also be a case to improve the possibilities for learning across sites, for example by connecting farmer networks across communities and possibly countries.

### Integrate Traders Better in Disease Management

Although traders are usually not the target for capacity development on infectious disease management in village poultry systems, the results from our study will suggest that traders have real reasons to engage in activities that will ensure farmers can obtain and use good quality vaccines, veterinary drugs, and other inputs they need. Traders and aggregators were found to handle live birds for considerable amounts of time and suffer substantial economic loss during disease outbreaks. Support to traders could be in capacity development for animal disease management but could also be more market oriented. Business models targeted to ensuring the delivery of veterinary inputs to smallholder producers will benefit from accounting correctly for the incentives of live bird traders in the system. Strategies to involve traders in the supply chain for veterinary inputs or services will however need to be well designed as traders in some communities were already perceived to hold unbalanced power. Care may need to be taken that traders are not in addition perceived to be displacing primary producers.

### Expand Scope for Private Sector and Market Opportunities

As with other studies addressing historical and institutional failures, many of the solutions directly proffered by the FGD respondents pointed to a larger role for the government. There may however be little appetite, despite indications of positive benefits to adopting communities (3) for increased public spending on wide-scale vaccination or similar programs to improve animal health care delivery to village poultry systems. It becomes imperative to explore the potential for private sector involvement, including the incentives for end users to take up the costs and/or coordination of vaccine and other veterinary input supply and use in village systems. In the case of *peste des petite ruminant* (PPR), a viral disease of small ruminant animals affecting mainly smallholders, a recent study showed that better communication on vaccine benefits through targeted information dissemination, and timely availability of vaccines with assured quality increased the willingness of farmers to vaccinate or pay for vaccines (44). Similar assessments will need to be completed to better understand what the cost implications will be for meaningful private sector engagement in the study communities in Ghana and Tanzania, and what incentives exist (and for whom) within the system.

In addition, interventions are needed, including business models, that improve the incentives for various value chain actors to adopt effective disease management within processes and outcomes that they control. Introducing mechanisms for livestock traceability should improve quality control in the village poultry system. This potentially could address concerns about, for example, local farmers selling off diseased birds to unsuspecting buyers. Traceability has however been a difficult concept to concretize in low resource agricultural and food systems, particularly outside of export value chains. In the context of the village poultry systems, the infrastructure that will be required to control, regulate, or monitor chicken production, sales or slaughter at the individual farmer level may not currently exist. However, early studies show promise of the presence of demand- side incentives to produce better quality animals and livestock products in low income countries, and work is ongoing to better understand what regulatory mechanisms align well with such incentives (45).

## Conclusion and Next Steps

Vaccines and veterinary drugs for the management of common infectious poultry diseases such as ND, are largely effective and readily available in urban areas and at affordable prices. However, long distances, poor infrastructure, and low business potential in rural areas prevent private suppliers from investing in these areas where most poultry producers reside. Inadequate education and training on the part of the producers reduces effectiveness of disease preventative and treatment options when they are available. Economic (e.g., lack of transportation means) and social prohibitions (e.g., restrictions on the engagement of women in certain economic activities) further limit the access of many women to veterinary inputs and services.

Poultry health care delivery options with high chances of success in the studied communities will be those that focus mainly on the delivery of quality veterinary products and services that are affordable, enhance supply of quality drugs and vaccines in rural areas and are tailored to reach poorer producers and more women by e.g., compensating for their reduced mobility, and access to information and markets. They also need to address trader as much as farmer/producer concerns. To improve management of infectious poultry diseases in developing countries, systems of veterinary inputs and services provision can build on what exists while better taking farmer needs and perceptions into consideration. Community-based approaches and increased use of technology such as mobile phones have much to offer, as do increased engagement and cooperation between government, non-governmental organizations, private sector, and cultural institutions, and appropriate investments by private enterprise and farmers themselves.

Our study investigating the perceptions of nearly 1,000 farmers and other value chain actors in Ghana and Tanzania, on interventions needed to address animal health challenges in village poultry systems largely agrees with the literature. This study highlights the need to expand coverage to better reach women, particularly as they are the majority of small-scale chicken farmers, and emphasizes the need for solutions to include others such as traders that are usually not involved throughout the production process but are very much adversely affected by the outcomes of poor poultry health at farm level. It also calls attention to heightened challenges that rural chicken producing communities are up against that have lower literacy rates and/or more limited access to information and communications technologies compared to other poor communities. A new project in Ghana is testing approaches to support women animal health service providers in chickens (and goats) as a way of reaching women farmers^1^. The project is adopting a transformative approach that aims to address some of the gender norms that the respondents of this study mentioned as limiting their ability to effectively raise chickens.

New quantitative analyses are needed to understand (1) what types of producers will be willing to pay market prices for improved veterinary inputs and services (e.g., using consumer choice experiments)—we found divergences in thought about whether the farmers and other value chain actors should bear full or partial costs of vaccines and other inputs; (2) to what extent willingness to pay on the part of users will incentivize capital inflows from private providers of vet inputs and services; (3) what society stands to gain by investing in village chicken production vs. other candidates for public investment; and (4) what policy or other government initiatives are needed.

## Data Availability Statement

The raw data supporting the conclusions of this article will be made available by the authors, without undue reservation.

## Ethics Statement

The studies involving human participants were reviewed and approved by the Ethics Committee for Basic and Applied Sciences (ECBAS), University of Ghana Ref. No. ECBAS046/18-19. Ethical clearance was obtained in Tanzania from the Office of the Vice-Chancellor, Sokoine University of Agriculture, and research permits for Singida (Ref. No. DPRTC/R/142/Vol. I /42) and Dodoma (Ref. No. DPRTC/R/142Vol. I/43) districts. Informed written consents were obtained from all participants in the study. The participants provided their written informed consent to participate in this study.

## Author Contributions

DE led the design and writing of the manuscript and contributed to the data analysis. EO and AG contributed to the design of the study, analysis of the data, and writing of the manuscript. TK, YA, GC, JK, FAM, FM, and MD contributed to the writing. JK and HJ assisted with the qualitative analysis. All authors reviewed and revised the manuscript.

## Conflict of Interest

The authors declare that the research was conducted in the absence of any commercial or financial relationships that could be construed as a potential conflict of interest.

## Footnotes

^1^https://www.ilri.org/news/annual-report/ghana-why-the-goats-will-not-die-on-her-watch, accessed 28 September 2020.
